# Cephalalgia—A “Tricky” Symptom for the Cardiologist: Acute Coronary Syndrome Presenting as Headache

**DOI:** 10.1002/ccr3.72066

**Published:** 2026-02-23

**Authors:** Alexandros Vassiliades, Varnavas Dimitriades, Vasiliki Vanesa Stylianou, Marios Pavlou, Panagiota Georgiou, Lorentzos Kapetis, Dimitrios Patestos, Michalis Tsielepis, George Bazoukis

**Affiliations:** ^1^ Department of Cardiology Larnaca General Hospital, State Health Services Organization Larnaca Cyprus; ^2^ Department of Basic and Clinical Sciences, University of Nicosia, Medical School, Nicosia Cyprus

**Keywords:** acute coronary syndrome, cephalalgia, headache, myocardial ischemia

## Abstract

Rarely, headache may be the primary or sole symptom of acute myocardial ischemia; clinicians should recognize such atypical presentations and obtain an electrocardiogram and cardiac biomarkers to establish the diagnosis and guide urgent treatment.

## 1 Clinical Case

**FIGURE 1:**
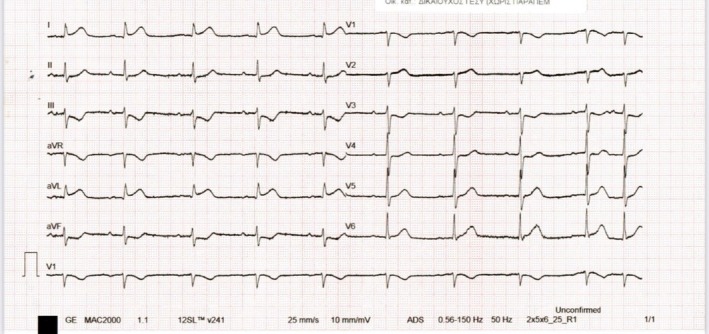
Electrocardiogram (ECG) of the patient during the initial presentation at the emergency department. The ECG shows ST‐segment elevation in leads I and aVL with reciprocal ST‐segment depression in leads II, III, and aVF.

**FIGURE 2:**
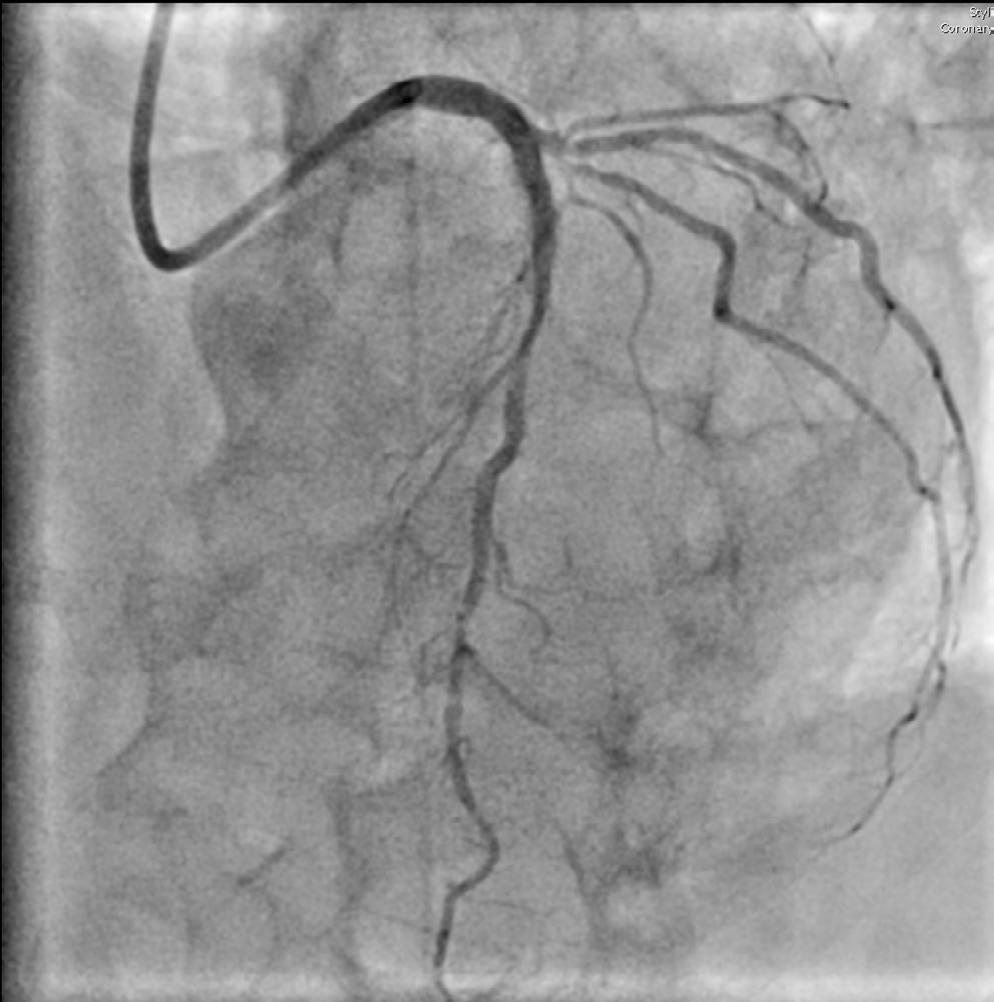
Coronary angiography revealed a significant stenosis of the first diagonal branch, which was identified as the culprit lesion.

**FIGURE 3:**
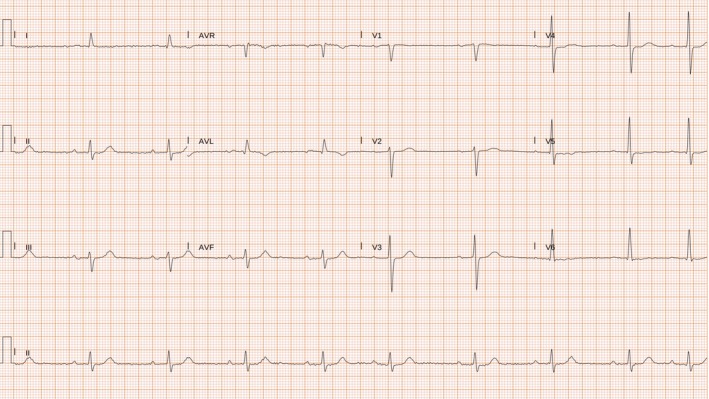
Electrocardiogram obtained 1 month after cardiac catheterization shows nonspecific ST–T–wave abnormalities in the lateral leads.

An 84‐year‐old patient presented to the emergency department with a recent‐onset headache that began approximately 3 h before arrival. The headache was notable for awakening the patient from sleep and was described as unlike any previous headaches. The headache was exertion‐related and accompanied by nausea but not photophobia. Past medical history included coronary artery disease with percutaneous coronary intervention (PCI) to the left main coronary artery (LMCA)–left anterior descending (LAD) and right coronary artery (RCA) in 2020, arterial hypertension, and dyslipidemia. Physical examination was unremarkable. A brain computed tomography (CT) scan was performed, which excluded intracranial pathology. An electrocardiogram (ECG) showed ST‐segment elevation in leads I and aVL with reciprocal ST‐segment depression in leads II, III, and aVF (Figure 1). Serum troponin levels were markedly elevated at 38 ng/mL (normal < 0.10 ng/mL). Interestingly, improvement of the headache was associated with normalization of the ECG abnormalities. The echocardiogram showed a preserved left ventricular ejection fraction with no regional wall motion abnormalities. Coronary angiography revealed a significant stenosis of the first diagonal branch, which was identified as the culprit lesion (Figure 2). PCI was successfully performed using a drug‐coated balloon. No recurrence of headache occurred following successful revascularization. An ECG obtained 1 month after PCI showed nonspecific ST‐T abnormalities in the lateral leads (Figure 3).

## 2 Discussion

This case highlights an atypical presentation of acute coronary syndrome, manifesting primarily as a headache in an elderly patient. Cardiac cephalalgia has been described in the literature as an ischemic equivalent similar to angina pectoris [1]. According to the International Classification of Headache Disorders (ICHD‐3), cardiac cephalalgia is defined as a migraine‐like headache that is usually, but not always, triggered by exertion, occurs in the context of myocardial ischemia, and is relieved by nitrates [2]. Key clinical features favoring cardiac cephalalgia compared to other causes of headache include: (a) new‐onset headache in older patients or in patients with cardiovascular risk factors, (b) headache triggered by exertion or emotional stress, (c) headache that resolves with nitrates or revascularization, (d) pain associated with ECG abnormalities or a rise in troponin levels, and (e) normal neurological examination and brain imaging.

In our patient, the presence of ECG changes, the absence of focal neurological deficits, the normal CT scan of the brain, and normalization of the ECG alongside improvement of the headache supported the diagnosis of cardiac cephalalgia. Although the pathophysiological mechanisms of cardiac cephalalgia are not fully understood, some mechanisms have been proposed. One suggests that the release of proinflammatory neurotransmitters such as bradykinin, histamine, adenosine, and serotonin during myocardial ischemia may cause vasodilation and subsequent headache [3]. Another mechanism includes convergence of afferent cardiac vagal fibers and afferent pathways of cranial pain fibers [3]. In conclusion, although headache is a rare symptom of acute myocardial ischemia, clinicians should be aware of atypical presentations of acute coronary syndromes to provide appropriate management for these patients. Misdiagnosis of acute coronary syndrome may result in delayed reperfusion and an increased risk of malignant arrhythmias, heart failure, and ultimately higher morbidity and mortality.

## Author Contributions

**Alexandros Vassiliades:** writing – review and editing. **Varnavas Dimitriades:** writing – review and editing. **Vasiliki Vanesa Stylianou:** writing – review and editing. **Marios Pavlou:** writing – review and editing. **Panagiota Georgiou:** writing – review and editing. **Lorentzos Kapetis:** writing – review and editing. **Dimitrios Patestos:** writing – review and editing. **Michalis Tsielepis:** writing – review and editing. **George Bazoukis:** supervision, writing – review and editing.

## Funding

The authors have nothing to report.

## Consent

The authors have obtained written informed consent from the patient.

## Conflicts of Interest

The authors declare no conflicts of interest.

## Data Availability

The authors have nothing to report.
